# LBH589 reduces oxidized mitochondrial DNA and suppresses NLRP3 inflammasome activation to relieve pulmonary inflammation

**DOI:** 10.1371/journal.pone.0328522

**Published:** 2025-08-04

**Authors:** Changwen Ning, Fenghua Gao, Zhe Wang, Huaying An, Pengyu Liu, Yanan Sun, Ru Li, Zhuyang Song, Yuanyuan Yuan, Jinlong Li, Jun Ma, Xingwei Jiang, Qun Yu

**Affiliations:** Institute of Health Service and Transfusion Medicine, Academy of Military Medical Sciences, Beijing, China; Chung Shan Medical University, TAIWAN

## Abstract

The NOD-like receptor protein (NLRP)3 inflammasome plays a critical role in acute respiratory distress syndrome (ARDS) by activating caspase-1, which cleaves the precursor forms of IL-1β and IL-18 into active cytokines and induces pyroptosis by cleaving gasdermin D (GSDMD). LBH589, a pan-histone deacetylase inhibitor, exhibits promising anti-inflammatory and immunomodulatory properties. However, the protective effect and underlying mechanism of LBH589 against ARDS is still unclear. In this study, we aim to determine whether and how LBH589 inhibits NLRP3 inﬂammasome activation while exerting its anti-inﬂammatory effect. Our data demonstrated that LBH589 effectively suppressed NLRP3 inflammasome activation in lipopolysaccharides (LPS)-primed and adenosine triphosphate (ATP)-stimulated J774A.1 cells and bone marrow-derived macrophages (BMDMs), evidenced by attenuated cleaved caspase-1 and IL-1β, IL-18, IL-16 release, as along with reduced GSDMD-mediated pyroptosis and ASC speck formation. Additionally, LBH589 significantly decreased mitochondrial reactive oxygen species (mtROS) and oxidized mitochondrial DNA (Ox-mtDNA), key triggers of inflammasome activation. Importantly, both prophylactic and therapeutic administration of LBH589 inhibited the pro-inflammatory cytokines secretion in lung tissue and ameliorated lipopolysaccharide (LPS)-induced ARDS in mice. These findings suggest that LBH589 may provide therapeutic benefits in ARDS by attenuating NLRP3 inflammasome activation and pyroptosis.

## Introduction

Acute respiratory distress syndrome (ARDS) is characterized by an acute and diffuse inflammatory injury to the lungs, which is triggered by severe infections and traumatic events [1]. About 8–10% of patients admitted to intensive care units (ICU) are diagnosed with ARDS and 24% of these patients require mechanical ventilation. The mortality rate of patients with ARDS is high and ranges from 35% to 50% [2,3]. The onset of ARDS is linked to uncontrolled lung inﬂammation and tissue injury driven by the activity of the innate immune cells, monocytes, macrophages, and neutrophils through secretion of interleukin (IL)-1β, IL-18, IL-6, and tumor necrosis factor (TNF). This is manifested by respiratory distress, refractory hypoxemia, and respiratory failure [1,4]. Currently, ARDS-specific drugs have not been approved for clinical treatment [5]. Therefore, clinical management of ARDS patients involves supportive measures such as mechanical ventilation, fluid management, lung-protective ventilation, prone positioning, recruitment maneuvers, and extracorporeal membrane oxygenation (ECMO) [2,6–8]. However, mechanical ventilation can adversely affect patient recovery by triggering airway damage and pulmonary complications. Furthermore, although venous ECMO is life-saving in severe cases, it is resource-intensive with low certainty of evidence, thereby limiting its accessibility and standardized application [9]. Current medications such as steroids and respiratory dilators have limited efficacy in controlling inflammation and may lead to side effects [9,10]. Emerging therapies, such as stem cell therapies, are promising but face hurdles in clinical translation because of safety concerns and heterogeneous patient responses [11]. Therefore, there is an urgent need for exploration of effective therapeutic modalities.

The NOD-like receptor pyrin domain-containing protein 3 (NLRP3) inflammasome plays a pivotal role in the pathogenesis of ARDS. The NLRP3 inﬂammasome is a multiprotein complex composed of NLRP3 (the sensor), caspase-1 (the enzyme), NEK7 (the mitotic kinase), and ASC (the scaffold protein), and is ubiquitously expressed in several immune cells, including macrophages, granulocytes, antigen-presenting cells (APCs), as well as T and B lymphocytes [12]. Activation of NLRP3 inflammasome is a two-step process involving the priming and activation signals. TLR agonists such as LPS firstly stimulates the NF-κB pathway to promote NLRP3, pro-IL-1β and pro- IL-18 expression. Then, the assembly of the NLRP3 inflammasome is triggered by pattern recognition receptors (PRRs), which detect extracellular pathogen-associated molecular patterns (PAMPs) and endogenous damage-associated molecular patterns (DAMPs), leading to its activation [13,14]. When primed, assembly of this complex facilitates autocatalytic cleavage of pro-caspase-1 into its active dimeric form (p20/p10). The activated caspase-1 subsequently converts the precursor forms of IL-1β and IL-18 into their mature forms. Concurrently, active caspase-1 cleaves gasdermin D (GSDMD) at a specific site to generate the N-terminal fragment (GSDMD-NT), which interacts with phospholipids on the cytoplasmic side of the plasma membrane and forms pores with diameters ranging from 12 to 14 nm. The pores facilitate secretion of IL-1β and IL-18 and initiate the inflammatory form of cell death called pyroptosis. IL-1β and IL-18 are the hallmark pro-inflammatory cytokines of the IL-1 family that enhance immune responses under both acute and chronic inflammatory conditions by recruiting neutrophils and macrophages [15–19]. Cytokines regulated by the inflammasome are associated with poorer clinical outcomes in ARDS patients [20].

Mitochondrial DNA (mtDNA) is a closed circular double-stranded DNA molecule located within the mitochondria and functions as a damage-associated molecular pattern (DAMP) that can induce inflammatory responses [21,22]. Cytidine/uridine monophosphate kinase 2 (CMPK2) is a rate-limiting enzyme for the synthesis of deoxyribonucleoside triphosphates. Therefore, it regulates the rate of mtDNA replication [23]. Factors such as ATP, monosodium urate (MSU) crystals, and other DAMPs contribute to mitochondrial damage and the subsequent production of reactive oxygen species (ROS) [23]. Mitochondrial ROS (mtROS) does not directly activate the NLRP3 inflammasome, but mtDNA oxidized by mtROS (Ox-mtDNA) can directly induce the NLRP3 inﬂammasome activation [21,23]. This activation prompts the formation of GSDMD pores in the membranes of peripheral immune cells, including the monocytes, macrophages, and neutrophils, thereby releasing excessive amounts of Ox-mtDNA into circulation and initiating the inﬂammatory cascade [21,24,25]. Because of limited mtDNA repair capabilities and the absence of protective histone proteins [26], it is highly vulnerable to oxidation, especially the formation of 8-hydroxy-2-deoxyguanosine (8-OHdG) [23]. However, it is not clear whether inhibiting the generation of Ox-mtDNA is an effective therapeutic strategy for ARDS.

LBH589 (Panobinostat), a potent pan-histone deacetylase (HDAC) inhibitor, has been approved by the United States Food and Drug Administration (FDA) for clinical use in the treatment of multiple myeloma [27,28]. Previous studies have demonstrated that histone deacetylase (HDAC) inhibitors possess significant anti-inflammatory properties [29]. LBH589 has also shown potential in the treatment of conditions such as gout and osteoarthritis [30,31]. Despite the emerging recognition of anti-inflammatory activity of LBH589, the detailed mechanism of LBH589 regulating NLRP3 inflammasome and pyroptosis has not been fully clarified. Furthermore, the effects of LBH589 on ARDS have not been characterized previously. In this study, we demonstrated that LBH589 inhibited NLRP3 inflammasome activation and pyroptosis, which was accompanied by the reduction in caspase-1 cleavage, mature IL-1β/18 release, and formation of ASC specks. The inhibitory effect of LBH589 treatment on NLRP3 inflammasome activation was mediated through decreased mtROS and Ox-mtDNA. We also demonstrated that LBH589 mitigated LPS-induced ARDS in mice. Our study suggests LBH589 can be a candidate for the treatment of ARDS and other NLRP3-related inflammatory diseases.

## Materials and methods

### Reagents

LBH589 and dexamethasone were obtained from Selleck Chemicals (Houston, USA). Lipopolysaccharide (LPS) (Escherichia coli O55:B5) and adenosine disodium triphosphate hydrate (ATP disodium) were purchased from Sigma-Aldrich (St. Louis, USA). Anti-GSDMD antibody (ab209845) was bought from Abcam (Cambridge, United Kingdom). Anti-caspase-1 (p20) antibody (AG-20B-0042) and anti-NLRP3 antibody (AG-20B-0014) were sourced from Adipogen AG (Liestal, Switzerland). Goat anti-rabbit antibody (AS014) and α-Tubulin (AC012) was obtained from ABclonal (Wuhan, China), and rabbit anti-8-OHdG was acquired from Bioss (Beijing, China). The following antibodies were sourced from Cell Signaling Technology (CST, Danvers, USA): β-actin (8H10D10), GAPDH (14C10), ASC (#67824), NF-κB p65 (D14E12), p-NF-κB p65(Ser536) (93HI), goat anti-mouse antibody (7076S), and Alexa Fluor 488 goat anti-rabbit IgG (#4412).

### Cell culture and stimulation

The J774A.1 cell line was purchased from the American Type Culture Collection (ATCC, Manassas, USA) and cultured in the Dulbecco’s modified Eagle’s medium (DMEM) (Gibco, Australia) supplemented with 10% fetal bovine serum (FBS) (Ex Cell Bio, USA) in a humidified incubator maintained at 5% CO_2_ and 37°C. To activate the NLRP3 inflammasome, J774A.1 cells were seeded in 24-well or 6-well plates and incubated overnight under standard conditions. Then, the culture medium was replaced with fresh DMEM containing 10% FBS. The cells were treated with 1 µg/ml LPS for 5 h with or without LBH589 (25, 50, 100 nM), and stimulated with ATP for 1h.

### Cell viability assay

Cell viability was assessed using the cell counting kit-8 (CCK-8) assay (Dojindo Laboratories, Beijing, China). J774A.1 cells (4 × 10^4 cells/well) were cultured in a 96-well plate and treated with LBH589 (25−100 nM) for 12 h. Then, the cells were further incubated for 2 h at 37°C after adding 10 μl of CCK-8 working solution to each well. The optical density of each well was then measured at 450 nm using a microplate reader (Tecan Spark, Switzerland).

### Enzyme-linked immunosorbent assay (ELISA)

The IL-6, IL-1β and TNFα in the culture supernatants were analyzed using the Mouse ELISA kits (DAKEWE, Shenzhen, China) and IL-18 were analyzed using the Mouse ELISA kits (CUSABIO, Wuhan, China) according to the manufacturer’s instructions. The concentrations of inflammatory cytokines were measured at an absorbance wavelength of 450 nm.

### Western blotting

Whole cell lysates were harvested using the cell lysis buffer (Solarbio, Beijing, China). Total protein concentration was determined using the BCA Protein Assay Kit (Thermo Fisher Scientific, Rockford, USA). Equal concentrations of whole-cell protein lysates were separated by SDS-polyacrylamide gel electrophoresis (SDS-PAGE) and transferred electrophoretically onto polyvinylidene difluoride (PVDF) membranes (Millipore Corporation, Billerica, USA). The membranes were blocked with 5% skimmed milk for 1 h. Then, the membranes were incubated overnight at 4°C with primary antibodies (1:1000) against NLRP3, ASC, GSDMD, caspase-1 (p20), and α-Tubulin. Subsequently, the membranes were incubated with HRP-conjugated secondary antibodies for 1 h. The blots were developed with the enhanced chemiluminescence kit (Millipore Corporation, Billerica, USA), and the protein bands were visualized and photographed using a Biomolecular Imaging System (GE ImageQuant LAS 500, USA).

### Double staining with Hochest33342 and PI

Inflammatory cell death was detected using the Hochest33342 and propidium iodide (PI) double staining assay kit (Solarbio, Beijing, China). J774A.1 cells (1 × 10^6 cells/well) were cultured overnight in a 6-well plate and treated with 1 µg/ml LPS for 5 h with or without LBH589. The cells were then stimulated with ATP for 1 h and stained with PI (2 µg/ml) and Hoechst 33342 (5 µg/ml) for 20 minutes at 4°C. The stained cells were observed and photographed using a fluorescence inverted microscope (ZEISS Axio Vert.A1, Germany) to document the proportion of cell death. Data were representative of at least six randomly selected image fields. PI-positivity indicated cell death.

### LDH assay

Lactate dehydrogenase (LDH) activity in the cell supernatants was assessed using the LDH Cytotoxicity Assay Kit (Beyotime Biotechnology, Shanghai, China). Briefly, J774A.1 cells were seeded in a 6-well plate and incubated overnight and treated with 1 µg/ml LPS for 5 h with or without LBH589. Afterward, the cells were stimulated with ATP for 1 h and the supernatants were collected. Then, in a 96-well plate, 120 μl of the culture supernatant was incubated with 60 μl of the working LDH assay solution for 30 minutes at room temperature in the dark. Absorbance was measured at 490 nm using a microplate reader (Tecan Spark, Switzerland). Cytotoxicity was calculated according to the manufacturer’s instructions.

### ASC oligomerization and ASC speck formation

J774A.1 cells were seeded in a 6-well plate and incubated overnight before the treatments described above. Following treatment, the supernatants were removed and cells were lysed using ice-cold PBS containing 0.5% Triton-X 100 for 30 min. The lysates were then centrifuged at 6,000 × g for 15 min at 4°C. The pellets were washed once with 1 ml of ice-cold PBS and resuspended in 200 µl of PBS. To analyze ASC oligomerization, 2 mM disuccinimidyl suberate (DSS) was added to the resuspended pellets, and the mixture was incubated at room temperature for 30 minutes with gentle rotation. The samples were subsequently centrifuged at 6,000 × g for 15 min at 4°C. The cross-linked pellets were resuspended in the 1 × SDS loading buffer, boiled for 5 min, and analyzed by western blotting using an anti-ASC antibody.

To observe the formation of ASC specks, J774A.1 cells were subjected to different treatments as described previously. Then, they were fixed in 4% paraformaldehyde for 15 min and permeabilized with ice-cold PBS containing 0.5% Triton-X 100 for 5 minutes. After permeabilization, the cells were blocked with 2% BSA for 30 min. Subsequently, cells were incubated with primary antibodies at 4°C overnight. Then, the cells were incubated with the Alexa Fluor 488-conjugated goat anti-rabbit IgG. The nuclei were stained with Hoechst 33342. Fluorescence images were acquired by Zeiss Axio Vert A1 fluorescent microscope.

### Real-time quantitative reverse transcription PCR (RT-qPCR)

J774A.1 cells (1 × 10^6 cells/well) were seeded in a 6-well plate and treated as previously described. Then, total RNA was extracted from the cells using TRIzol (TIANGEN, Beijing, China). Complementary DNA (cDNA) was synthesized from 2 µg of total RNA using the All-In-One 5X RT MasterMix for qPCR kit (Abm, Hercules, Canada). The cDNA samples were then subjected to real-time qPCR using the UltraSYBR mixture (CWBIO, Beijing, China) in a LightCycler® 96 Real-Time PCR Machine (Roche, Switzerland) according to the manufacturer’s instructions. The expression levels of mRNAs were analyzed relative to the GAPDH mRNA levels using the 2^-∆Ct^ method. The primer sequences for RT-qPCR are listed in Table 1.

**Table 1:** Primers used in RT-qPCR.

Genes	Primers for real time-PCR	
Forward (5′–3′)	Reverse (5′–3′)
*Il-1β*	GCCCATCCTCTGTGACTCA	AGGCCACAGGTATTTTGTC
*Il-18*	TCCAACTGCAGACTGGCAC	CTGATGCTGGAGGTTGCAGA
*Il-6*	CCAGGTAGCTATGGTACTCCA	GCTACCAAACTGGATATAATC
*Tnfα*	CCCTCACACTCAGATCATCTT	GCTACGACGTGGGCTACAG
*Ccl2*	GCTACAAGAGGATCACCAGCAG	GTCTGGACCCATTCCTTCTTGG
*Ccl3*	ACTGCCTGCTGCTTCTCCTACA	ATGACACCTGGCTGGGAG
*Ccl4*	ACCCTCCCACTTCCTGCTGTTT	CTGTCTGCCTCTTTTGGTCAGG
*Cmpk2*	TGGTAAGACCACACTGACGC	GCTGTGCTATGCCAGTACCT
*Gapdh*	TCTTGGGCTACACTGAGGAC	CATACCAGGAAATGAGCTTGA

### Animal experiments and ethics statement

Male BALB/c mice (8−10 weeks old) were purchased from Vital River Laboratories (Beijing, China) and subjected to LPS-induced acute ARDS. The animal study was conducted according to the internationally recognized guidelines and approved by the ethics committee of the Beijing Institute of Transfusion Medicine (application number: IACUC-DWZX-2024–565). All mice were maintained in pathogen free cages at 24 °C under a 12-h light–dark cycle. The mice were provided free access to rodent diet and tap water and subjected to adaptive feeding for seven days.

LBH589’s prophylactic effect was evaluated via oral gavage at a dosage of 10 mg/kg for three consecutive days. The final dose was administered 2 h prior to the LPS challenge. The mice were administered an intra-peritoneal injection of 5 mg/kg LPS or vehicle control. After 24 hours, the mice were first anesthetized with isoflurane gas to relieve pain or discomfort and then euthanized by cervical dislocation and their lungs were harvested. LBH589’s therapeutic effect was studied by oral gavage at 10 mg/kg and dexamethasone (4 mg/kg) administered intraperitoneally as positive control. Treatments began 30 minutes after LPS administration, followed by a second dose 24 hours later. Lung tissue was collected 24 hours after the final drug dose. In all cases, at the end of experiment, whole lung weights were recorded. The ratio of lung weight to body weight was calculated to determine the success of the LPS-induced inflammation model and the therapeutic effects of LBH589. Lung tissue samples were immersed in saline for 24 h at −80 °C. Then, the supernatant was used to measure cytokine levels using the ELISA kits.

### Hematoxylin & eosin (H&E) and immunohistochemical staining

The lung tissue were harvested after sacrificing the mice and fixed in the 10% formalin for 24 h. The tissue were then embedded in paraffin, sectioned, and stained with H&E to evaluate gross morphology, lung damage, and inflammation. Lung sections were subjected to incubation with antibodies speciﬁc for F4/80 (macrophage marker). Pathological images were captured using the Olympus microscope (Olympus, Japan).

### Mitochondrial function

J774A.1 cells were seeded in a 6-well plate overnight and treated as described above. The cells were subsequently stained with MitoSOX (500 nM) (Thermo Fisher, California, USA) for 15 min and washed three times with PBS. The nucleus was stained with Hoechst 33342. Fluorescence images were acquired using the Zeiss Axio Vert A1 fluorescent microscope. To measure the oxidized mitochondrial DNA (Ox-mtDNA) levels, the 8-OHdG content of mtDNA was detected by immunofluorescence. The immunofluorescence procedure was performed similar to the detection of ASC specks.

### Statistical analysis

The data are represented as mean ± SEM of three independent trials unless otherwise specified. Statistical analysis was performed using the GraphPad Prism 9.5.0 software. Two-tailed Student’s t-test or Welch’s t-test was used for comparing the data between two groups. Mann-Whitney test was used for nonparametric tests. p < 0.05 was considered as statistically significant.

## Results

### LBH589 suppresses production of cytokines and chemokines

**Fig 1:**
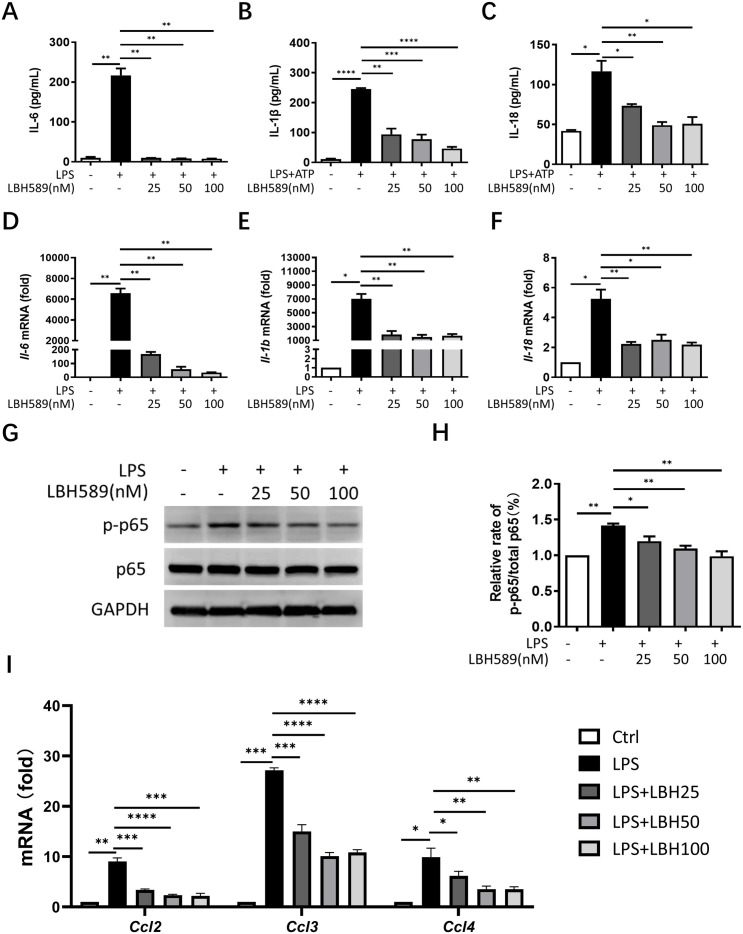
LBH589 suppresses the production of cytokines and chemokines. (A) J774A.1 cells were treated with LPS (1 µg/ml) with or without of LBH589 (25, 50, 100 nM) for 6 h or (B, C) the cells were treated with LPS (1 µg/ml) for 5 h in the presence or absence LBH589 and stimulated by ATP (5 mM) for 1 h. The release of IL-6, IL-1β and IL-18 in the supernatants were measured by ELISA. (D-F) The mRNA levels of *Il-1β*, *Il-6* and *Il-18* were detected by RT-qPCR. (G) J774A.1 cells were harvested 6 h after LPS stimulation with or without LBH589, p-p65 and total p65 levels were analyzed by western blot. GAPDH is used as internal control. (H) Quantitative analysis of the western blot results in G. The fold changes in the p-p65 and total p65 levels based on the densitometry measurements were normalized to GAPDH. (I) The relative mRNA levels of the indicated chemokines were detected by RT-qPCR. Results are shown as mean ± SEM (n = 3). ^*^indicates p < 0.05, ^**^indicates p < 0.01, ^***^indicates p < 0.001, ^****^p < 0.0001.

To understand the anti-inﬂammatory mechanisms of LBH589, we evaluated its effect on the activation of NF-κB and the expression levels of cytokines and chemokines in macrophages. J774A.1 cells and BMDMs were incubated with or without LBH589 and stimulated with LPS minus or plus ATP. Subsequently, cytokine levels in the culture supernatants were measured using ELISA. Our results indicated that LBH589 inhibited the secretion of IL-6, IL-1β, and IL-18 in both J774A.1 cells and BMDMs stimulated with LPS minus or plus ATP, but did not affect the secretion of TNF-α (Figs 1A–C, S1A, S2A–D). Consistent with the reduction in cytokines release, RT-qPCR analysis showed that LBH589 led to a significant decline in *Il-1β, Il-6* and *Il-18* mRNA induction by LPS (Figs 1D–F, S1B). LBH589 also suppressed LPS-induced p65/RelA phosphorylation. Relative p-p65 to total p65 amounts were decreased (Fig 1G, H). Furthermore, LBH589 reduced the mRNA levels of the NF-κB responsive chemokines, including *Ccl2, Ccl3,* and *Ccl4* (Fig 1I). These data suggest that LBH589 suppresses the expression of inﬂammatory cytokines and chemokines by inhibiting LPS-induced NF-κB activation.

### LBH589 represses activation of the NLRP3 inflammasome

**Fig 2:**
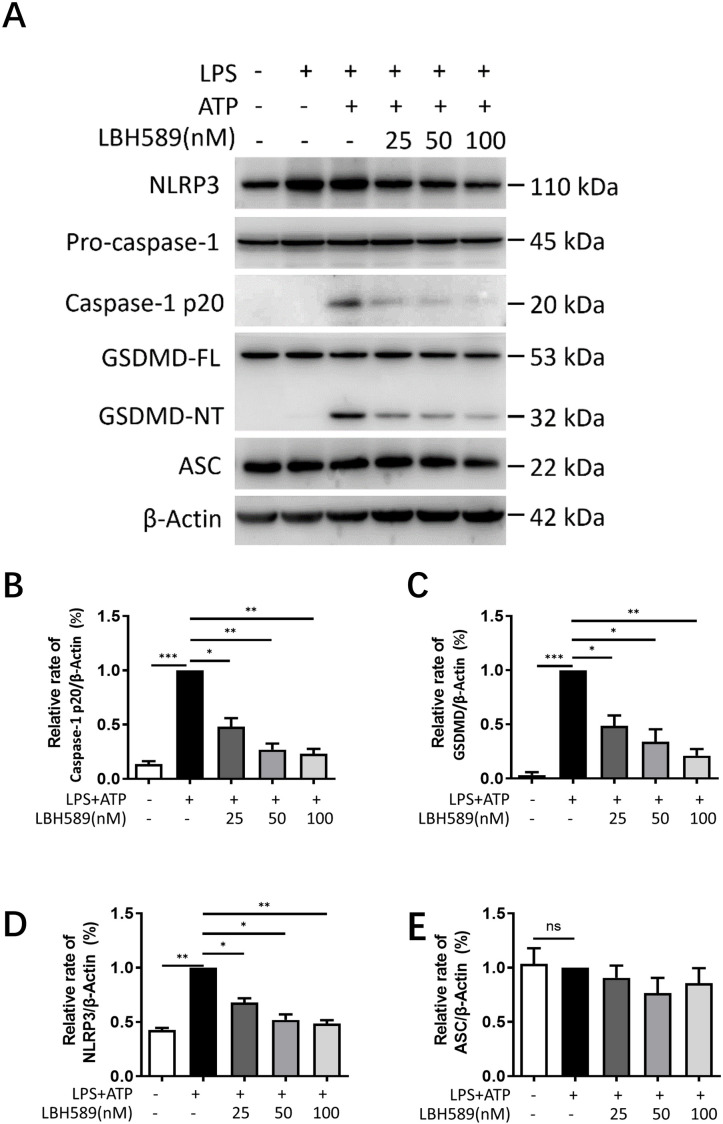
LBH589 inhibits NLRP3 inflammasome activation. J774A.1 cells were primed with LPS for 5 h in the presence or absence of LBH589 (25, 50, 100 nM), and then stimulated with ATP for 1 h. (A) Western blotting and subsequent quantification of (B) caspase‑1 p20, (C) GSDMD-NT, (D) NLRP3, (E) ASC protein levels. The fold changes based on the densitometry measurements were normalized to β-actin. Results are shown as mean ± SEM (n = 3). ^*^indicates p < 0.05, ^**^indicates p < 0.01, ^***^indicates p < 0.001, ns not significant.

Caspase-1 is an inflammatory caspase in the NLRP3 inflammasome that facilitates the release of mature IL-1β/18 and mediated inflammatory cell death named pyroptosis [16]. We analyzed whether LBH589 obstructed the cleavage of pro-caspase-1 by evaluating the effects of LBH589 in the LPS-primed and ATP stimulated macrophages. Immunoblotting results showed that caspase-1 p20 could be detected in LPS/ATP-stimulated J774A.1 cells and BMDMs, but LBH589 downregulated the levels of caspase-1 p20 under ATP stimulation (Figs 2A and B, S2E). Activated caspase-1 cleaves GSDMD to generate the GSDMD-NT fragment, which performs as an effector protein for pyroptosis [18]. Western blot analysis showed that LBH589 dose-dependently reduced the generation of GSDMD-NT in the LPS/ATP-activated macrophages (Figs 2A and C, S2E). Since NLRP3 inﬂammasome is involved in the activation of caspase-1 and is a multi-protein complex consisting of NLRP3, ASC, and pro-caspase-1, we evaluated the expression levels of NLRP3 and ASC. Our results showed that LBH589 treatment reduced the levels of NLRP3 in the LPS/ATP-activated macrophages in a dose-dependent manner (Figs 2A and D, S2E), which correlated with the inhibition of NF-κB signaling. However, the expression of ASC has no signiﬁcant change in the absence or presence of stimulating with LPS/ATP and LBH589 treatment did not alter ASC protein levels (Figs 2A and E, S2E). Taken together, we conclude that LBH589 restrain LPS/ATP-induced NLRP3 inﬂammasome activation.

### LBH589 reduces pyroptosis upon NLRP3 inflammasome activation

**Fig 3:**
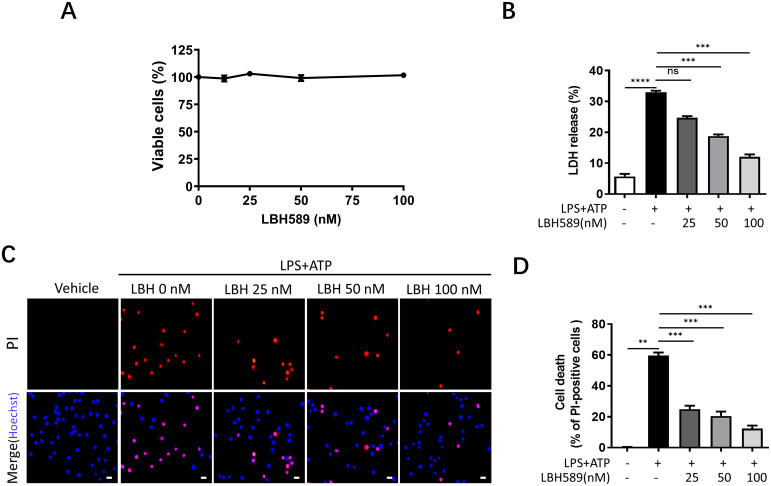
LBH589 restrains pyroptosis in J774A.1 cells. (A) J774A.1 cells were treated with vehicle control or variable concentrations of LBH589 in 96-well plates for 12 h and viable cells were determined using CCK-8 assays. (B) The cells were primed with LPS (1 µg/ml) for 5 h with or without LBH589, and then stimulated with ATP (5 mM) for 1h. The release of LDH in the supernatant was measured by LDH Cytotoxicity Assay Kit. Data are shown as mean ± SEM (n = 3). ^***^p < 0.001, ^****^p < 0.0001, ns not significant. (C) Representative immunofluorescence images of cell death were presented by PI and Hoechst 33342 staining. Scale, 20 μm. (D) The percentage of PI-positive cells relative to all was calculated; 6 randomly chosen fields were quantified. Data are shown as mean ± SEM (n = 6). ^**^p < 0.01, ^***^p < 0.001.

To investigate the effects of LBH589 on pyroptosis, we analyzed inflammatory cell death using the LDH cytotoxicity assay kit. We found that LBH589 dose-dependently restrained ATP-induced LDH release in J774A.1 and BMDMs (Figs 3B, S2F). We further analyzed inflammation mediator-induced cell death by staining the cells with propidium iodide (PI). The result showed that LBH589 decreased the ratio of PI-positive J774A.1 cells in a dose-dependent manner. (Fig 3C and D). We also used the CCK-8 cell viability assay to evaluate the cytotoxicity of LBH589 on J774A.1 cells. The data indicated that LBH589 did not exhibit significant cytotoxicity towards J774A.1 cells over a 12-hour treatment period at concentrations ranging from 0 to 100 nM (Fig 3A). Thus, these results suggest that LBH589 protects cells from pyroptosis upon NLRP3 inflammasome activation.

### LBH589 interferes with ASC assembly

**Fig 4:**
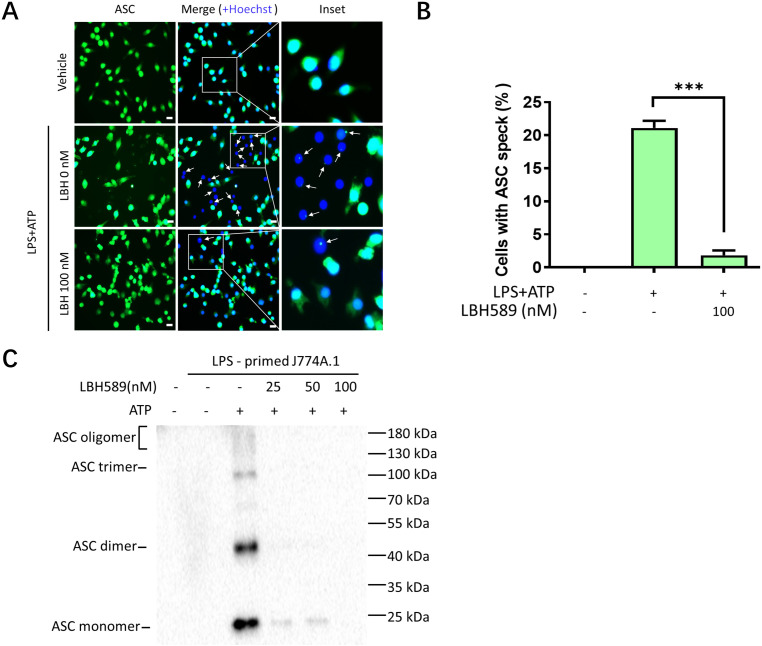
LBH589 blocks the assembly of ASC in J774A.1 cells. (A) Representative immunofluorescence images of ASC speck formation in LPS-primed J774A.1 and stimulated with ATP in the presence or absence of LBH589 (100 nM). White arrows indicate ASC specks (green). Scale bars, 20 μm. (B) Quantification of J774A.1 cells containing ASC speck formation in six random images. ^***^p < 0.001 versus inflammation-stimulated cells without LBH589 treatment. (C) ASC oligomerization in cross-linked cytosolic pellets was analyzed by immunoblotting.

During NLRP3 inflammasome activation, ASC protein oligomerizes with NLRP3 and pro-caspase-1 to form an active inflammasome complex. The assembly of ASC requires both the N-terminal pyrin domain and the C-terminal caspase recruitment domain (CARD). The pyrin domain facilitates the formation of filaments, whereas the CARD domain condenses these filaments into a concentrated structure known as ASC specks, which are hallmarks of inflammasome activation [32]. Our results demonstrated that LBH589 reduced the formation of ASC specks in LPS-primed J774A.1 cells under ATP stimulation conditions (Fig 4A, B). Furthermore, we used chemical cross-linking to assess ASC oligomerization, a process indicative of NLRP3 inflammasome activation. Immunoblotting results revealed that LBH589 dose-dependently reduced ATP-induced ASC oligomerization in LPS-primed J774A.1 cells (Fig 4C). Collectively, these findings further confirm that LBH589 disrupts NLRP3 inflammasome activation by obstructing ASC assembly, thereby preventing the formation of ASC specks in J774A.1 cells.

### LBH589 inhibits mtROS and Ox-mtDNA generation

**Fig 5:**
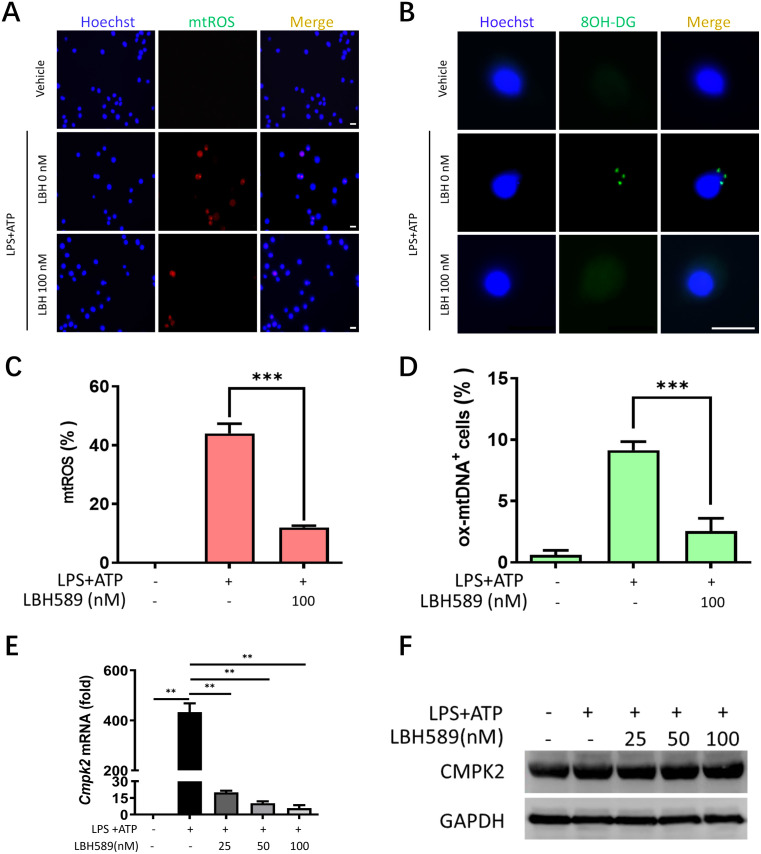
LBH589 inhibits mtROS and Ox-mtDNA generation. (A) Fluorescence microscopy for Mito-SOX Red-labelled ROS in cytosol of LPS-primed J774A.1 and stimulated with ATP in the presence or absence of LBH589 (100 nM). Scale bar, 20 µm. (B) Fluorescent microscopy images of J774A.1 cells that were stained with 8OH-dG. Scale, 20 μm. (C) Quantification of fluorescence density of mtROS. n = 6. ^***^p < 0.001 versus inflammation-stimulated cells without LBH589 treatment. Two-sided unpaired t test. (D) Quantification of cells with Ox-mtDNA. n = 6. ^***^p < 0.001 versus inflammation-stimulated cells without LBH589 treatment. (E) J774A.1 cells were primed with LPS for 5 h in the presence or absence of LBH589 (25, 50, 100 nM), and then stimulated with ATP for 1 h. The mRNA level of C*mpk2* was detected by RT-qPCR. Data are shown as mean ± SEM (n = 3). ^**^indicates p < 0.01. (F) Western blot analysis of CMPK2 protein expression.

Oxidative stress is caused by increased ROS production in the dysregulated mitochondria and is a common upstream signal for the activation of the NLRP3 inflammasome [23]. Therefore, we assessed mitochondrial ROS (mtROS) levels by staining the J774A.1 cells with MitoSOX, a dye that fluoresces upon oxidation by mtROS. Immunofluorescence analyses revealed that mtROS levels were increased in the LPS/ATP-activated J774A.1 cells, but treatment with LBH589 markedly blunted the levels of mtROS (Fig 5A, C). Mitochondrial ROS does not directly promote activation of the NLRP3 inflammasome, but oxidized mitochondrial DNA (Ox-mtDNA) that are generated by mtROS directly can activate the NLRP3 inflammasome [23]. Therefore, to further investigate the effects of LBH589 on the levels of oxidized mtDNA (Ox-mtDNA), we labeled oxidized DNA (marked with 8-hydroxy-2’-deoxyguanosine [8-OHdG]) in different group of J774A.1 cells. Our data showed that the levels of 8-OHdG were significantly increased in the LPS/ATP-stimulated J774A.1 cells compared to the untreated cell. However, LBH589 treatment diminished the synthesis of Ox-mtDNA in activated J774A.1 cells (Fig 5B, D). LBH589 treatment did not alter the protein expression levels of CMPK2 (Fig 5F), an LPS-inducible nucleotide kinase that converts dCMP to dCDP and regulates mtDNA synthesis. These results suggest that LBH589 modulates the activity of the NLRP3 inflammasome by regulating the levels of mtROS and Ox-mtDNA.

### LBH589 inhibits LPS-induced ARDS

In vivo, we investigated the effects of short-term LBH589 treatment on ARDS in a mouse model, which was generated by injecting a single bolus of 5 mg/kg LPS intraperitoneally. Initially, mice were given vehicle or 10 mg/kg LBH589 via oral gavage for three consecutive days with the last dose administered 2h prior to LPS challenge. Lung tissue were harvested 24 hours post-LPS injection and analyzed. Our findings indicated that LPS administration altered the alveolar structure with extensive infiltration of immune cells into the alveolar lumen and pronounced thickening of the interalveolar septa (S3A Fig). The lung index was notably increased compared to the untreated group, thereby confirming the successful establishment of the ARDS model mice (S3C Fig). Treatment with LBH589 protected against LPS-induced lung injury. Histological assessment of lung sections revealed that LBH589 treatment effectively prevented thickening of the alveolar septa and inhibited infiltration of macrophages into the lungs (S3A, B Fig). Furthermore, LBH589 treatment decreased the lung index (S3C Fig) and reduced the levels of IL-1β and IL-6 in the lung tissue (S3D and E Fig) compared with the corresponding controls.

**Fig 6:**
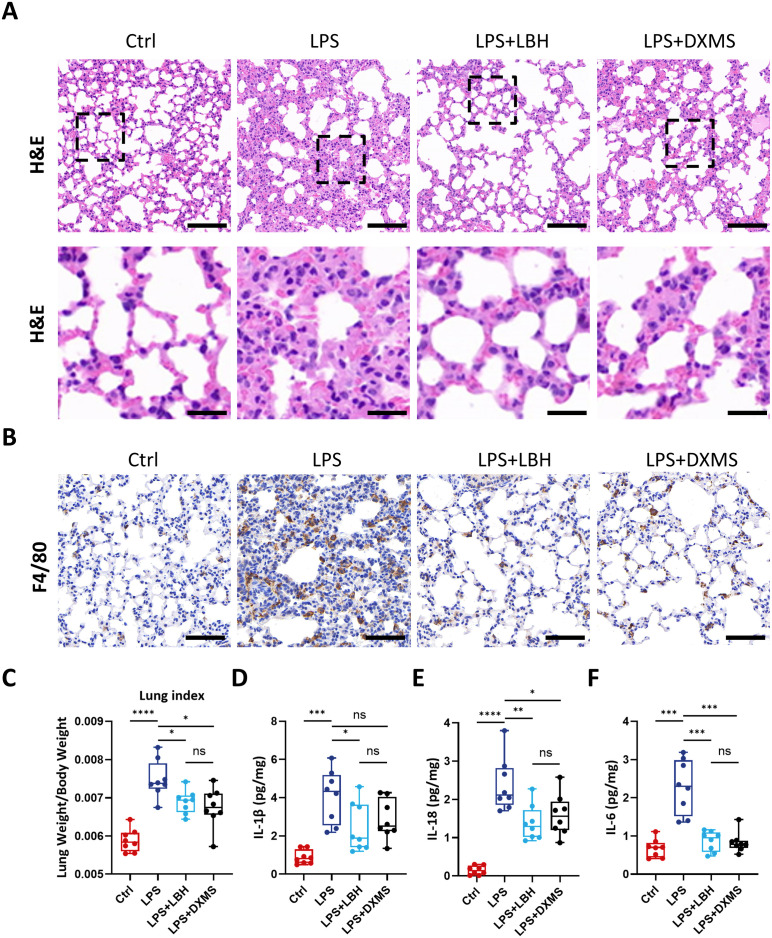
LBH589 has therapeutic effects on LPS-induced ARDS. (A) H&E of lung tissue from mice that were treated with vehicle or 10 mg/kg LBH589 daily starting 30 min after 5 mg/kg LPS challenge, and 4 mg/kg dexamethasone (DXMS) + LPS as positive control group. Tissue was collected 48 h after LPS administration. Scale bar, 100 μm and 25 μm; n = 8 mice per group. (B) Lung sections from above mice were stained with F4/80 antibodies. Scale bar, 50 μm. (C) The lung index was calculated as lung weight/body weight and graphed as mean ± SEM. IL-1β (D) IL-18 (E) and IL-6 (F) concentrations in lung tissue from above mice were measured by ELISA. ^*^ indicates p < 0.05, ^**^indicates p < 0.01, ^***^indicates p < 0.001, ^****^p < 0.0001, ns not significant.

Next, we tested whether LBH589 administration after LPS challenge ameliorated LPS-induced ARDS. Specifically, LBH589 was given via oral gavage at 10 mg/kg, 30 minutes after LPS injection, with a second dose administered after 24 hours. Dexamethasone served as a positive control. Mice were evaluated 24 hours after the final dose. Our results demonstrated that therapeutic administration of LBH589 was as effective as prophylactic treatment in reducing alveolar septal thickening (Fig 6A), macrophage infiltration (Fig 6B) and lung index (Fig 6C). Therapeutic LBH589 also decreased IL-1β, IL-18 and IL-6 (Fig 6D–F) levels in lung tissue. Although dexamethasone also exhibited protective effects and reduced cytokine production in lung tissue, no significant difference was observed between LBH589 and dexamethasone. Thus, our results suggest that LBH589 may be a potential candidate in the treatment of ARDS.

## Discussion

ARDS is a heterogenous syndrome with a complex clinical phenotype, leading to severe inflammation and cell death in the lungs [1,33]. Despite advances in treatment, the high mortality rate highlights the need for timely intervention to reduce inflammation and improve outcomes. Histone deacetylase inhibitors represent a promising class of drugs not only in cancer treatment [34,35] but also in the management of immune diseases [29]. LBH589 is a pan-HDAC inhibitor that is FDA-approved for treating multiple myeloma [27], which has shown potential anti-inflammatory and immunomodulatory actions [30,31]. However, whether short-term LBH589 treatment can attenuate acute inflammatory responses in ARDS remains unclear. Its clinical application is currently limited by the poorly understood mechanisms of action.

In this study, we administered LBH589 at a dose of 10 mg/kg, as used in preclinical antitumor studies in mice [36], and evaluated its preventive and therapeutic effects in an LPS-induced ARDS mouse model. Our data showed that short-term LBH589 administration effectively mitigated murine ARDS pathology, evidenced by reduced pulmonary edema, lung index, and suppressed the level of pro-inflammatory cytokines IL-1β/18 and IL-6 in lung tissue. These findings underscore its repurposing potential for this acute condition. Furthermore, LBH589 demonstrated protective effects whether administered preventively before or therapeutically after ARDS establishment, indicating flexibility in clinical scheduling. However, studies have indicated that chronic exposure to LBH589 at a dose of 20 mg/day orally can lead to hematologic adverse events in clinical trials involving combination therapies (NCT01242774, NCT02145715, clinicaltrials.gov). Although short-term administration of LBH589 may reduce the risk of toxic side effects, identifying the optimal dose and timing of anti-inflammatory treatments in humans to maximize benefits and minimize risks in ARDS remains crucial.

The NLRP3 inflammasome is crucial for host defense against pathogens, however, its abnormal activation can result in uncontrolled infections and contribute to metabolic, autoimmune, and neurodegenerative disorders [37]. During pyroptosis, activation of the classical NLRP3 inflammasome facilitates processing and activation of caspase-1. Subsequently, activated caspase-1 cleaves precursor IL-1β and IL-18 proteins into their mature forms and cleaves GSDMD into GSDMD-NT, thereby causing cellular damage and inflammatory cell death [18,19,38]. In this study, we demonstrated that LBH589 signiﬁcantly inhibit pro-inflammatory cytokines secretion, NLRP3 inﬂammasome activation, GSDMD-NT formation and pyroptosis in macrophages, indicating that LBH589 can be used as a novel candidate to treat NLRP3-driven diseases. Notably, a higher concentration of LBH589 (starting from 1 µM) was required in BMDMs to achieve effects comparable to those observed in J774A.1 cells (S2 Fig). The differences in drug sensitivity between the two cells may be attributed to several factors. J774A.1 cells, a murine macrophage-like cell line, may accumulate higher levels of the drug due to altered transporter activity compared to primary bone marrow-derived macrophages (BMDMs). Additionally, LBH589 is primarily metabolized by cytochrome P450 (CYP450) enzymes [39], and variations in enzyme activity between the cell types can influence the metabolism and thus the effective concentration of LBH589. ASC is a critical assembly protein of the NLRP3 inflammasome. Our findings demonstrated that LBH589 treatment did not alter ASC protein expression, but inhibited oligomerization of ASC (Fig 4). The oligomerization of ASC is regulated by post-translational modifications, including ubiquitination, deubiquitination, and phosphorylation [40,41]. Histone deacetylases not only regulate the acetylation of histones but also influence gene expression by targeting non-histone transcriptional regulators [34]. Hence, in the future, we aim to investigate the mechanism by which LBH589 affects ASC oligomerization, including analyzing whether this regulation is mediated through acetylation of ASC.

Activation of the NLRP3 inflammasome involves multiple upstream mechanisms. Previous studies have shown that reactive oxygen species (ROS) are one of the critical mediators of NLRP3 inflammasome activation [42]. It is postulated that ROS promote activation of the NLRP3 inflammasome by oxidizing mitochondrial DNA (mtDNA). Mitochondria are the primary sites of ROS production and the main target of ROS-induced damage. Excessive generation of mitochondrial ROS (mtROS) oxidizes the bases and induces strand breaks in the mitochondrial DNA, thereby disrupting the electron transport chain. Mitochondrial damage also leads to the release of free Ox-mtDNA into the cytoplasm, where it binds and activates the NLRP3 inflammasome [43]. Our data showed that LBH589 effectively inhibited mtROS production in the LPS/ATP-stimulated J774A.1 cells. We then assessed the levels of 8-OHdG, a biomarker used to assess oxidative damage to mtDNA [44,45]. Our results showed that LBH589 reduced the production of 8-OHdG in LPS/ATP-stimulated J774A.1 cells (Fig 5). CMPK2 is a rate-limiting enzyme involved in the synthesis of deoxynucleoside triphosphates and is critical for regulating mtDNA replication [44]. Our study showed that LBH589 significantly reduced the *Cmpk2* mRNA levels, but did not affect CMPK2 protein levels (Fig 5). This suggested the involvement of other mechanisms in the regulation of mtDNA by LBH589. Therefore, further studies are needed to characterize the specific mechanism by which LBH589 inhibits the accumulation of oxidized mtDNA.

It is important to note that, as a pan-HDAC inhibitor, LBH589 may also modulate ARDS through NLRP3 inflammasome-independent pathways, potentially leading to off-target effects. Different HDACs regulate distinct inflammatory responses by targeting specific signaling pathways and transcription factors. For instance, HDAC3 plays a crucial role in regulating multiple inflammatory signaling pathways such as NF-κB, MAPK, and JAK-STAT [46]. Similarly, HDAC6 controls cytokine signaling within the JAK/STAT pathway by deacetylating STAT3, thereby influencing cytokine-driven inflammation. [47]. Moreover, HDAC7 specifically interacts with key components of the TLR4 pathway, such as TRAF6 and TAK1, which are essential for propagating inflammatory signals [48]. To minimize potential off-target effects, we plan to utilize more selective HDAC inhibitors in future studies.

**Fig 7:**
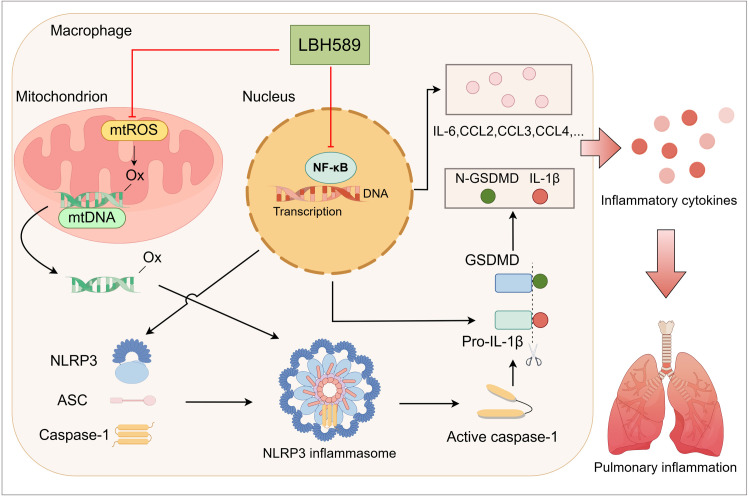
The molecular mechanism by which LBH589 inhibits NLRP3 inflammasome activation and pulmonary inﬂammation (By Figdraw).

In summary, LBH589 mitigates LPS-induced ARDS in mice by suppressing NLRP3 inflammasome activation and pyroptosis through reduction of mtROS and Ox-mtDNA (Fig 7). Therefore, LBH589 is a promising drug for treating ARDS and other NLRP3-related inflammatory diseases.

## Supporting information

S1 Fig Effect of LBH589 on TNFα, related to Fig 1. (A, B) J774A.1 cells were primed with LPS (1 µg/ml) for 6 h with or without LBH589. Supernatants were analyzed by ELISA for TNFα release in (A). The mRNA levels of *Tnfα* were detected by RT-qPCR in (B). Results are shown as mean ± SEM (n = 3). ^**^indicates p < 0.001, ns not significant.
(TIF)

S2 Fig LBH589 inhibited NLRP3 inflammasome activation and pyroptosis in BMDMs. (A-D) BMDMs were treated with LPS (100 ng/ml) in the presence or absence of LBH589 (1, 2, 4 µM) for 4 h and minus or plus ATP (4 mM) for 1 h. The release of IL-6, TNFα, IL-1β and IL-18 in the supernatants were measured by ELISA. (E, F) LPS-primed BMDMs were stimulated with ATP with or without LBH589. Cell extracts were analyzed by immunoblotting to NLRP3, caspase-1, GSDMD, ASC, α-Tubulin served as a loading control. Supernatants for LDH release assay are shown in (F). Results are shown as mean ± SEM (n = 3). ^*^indicates p < 0.05, ^**^indicates p < 0.01 and ^***^indicates p < 0.001, ^****^indicates p < 0.0001.
(TIF)

S3 Fig LBH589 has prophylactic effects on LPS-induced ARDS in mice. (A) H&E of lung tissue from mice that were pretreated with vehicle or 10 mg/kg LBH589 for three consecutive days and challenged with 5 mg/kg LPS 24 h prior tissue collection. Scale bar, 100 μm and 25 μm. n = 8 mice per group. (B) Lung sections from above mice were stained with F4/80 antibodies. Scale bar, 50 μm. (C) The lung index was calculated as lung weight/body weight and graphed as mean ± SEM. IL-1β (D) and IL-6 (E) concentrations in lung tissue from above mice were measured by ELISA. ^*^ indicates p < 0.05, ^**^indicates p < 0.01, ^***^indicates p < 0.001, ^****^p < 0.0001.
(TIF)

S1 Text BMDM culture and stimulation. (DOCX)

S1 Raw Images This file contains the original, uncropped and minimally adjusted images supporting all blot results reported in an article’s figures and supporting information files. (PDF)

S2 Raw Data This file contains the data required to replicate all study findings reported in the article. (XLSX)
