# Supplementary figures and images for: LBH589 reduces oxidized mitochondrial DNA and suppresses NLRP3 inflammasome activation to relieve pulmonary inflammation
Source: PLoS One. 2025 Aug 4;20(8):e0328522. doi: 10.1371/journal.pone.0328522 (PMC12321101; doi:10.1371/journal.pone.0328522)

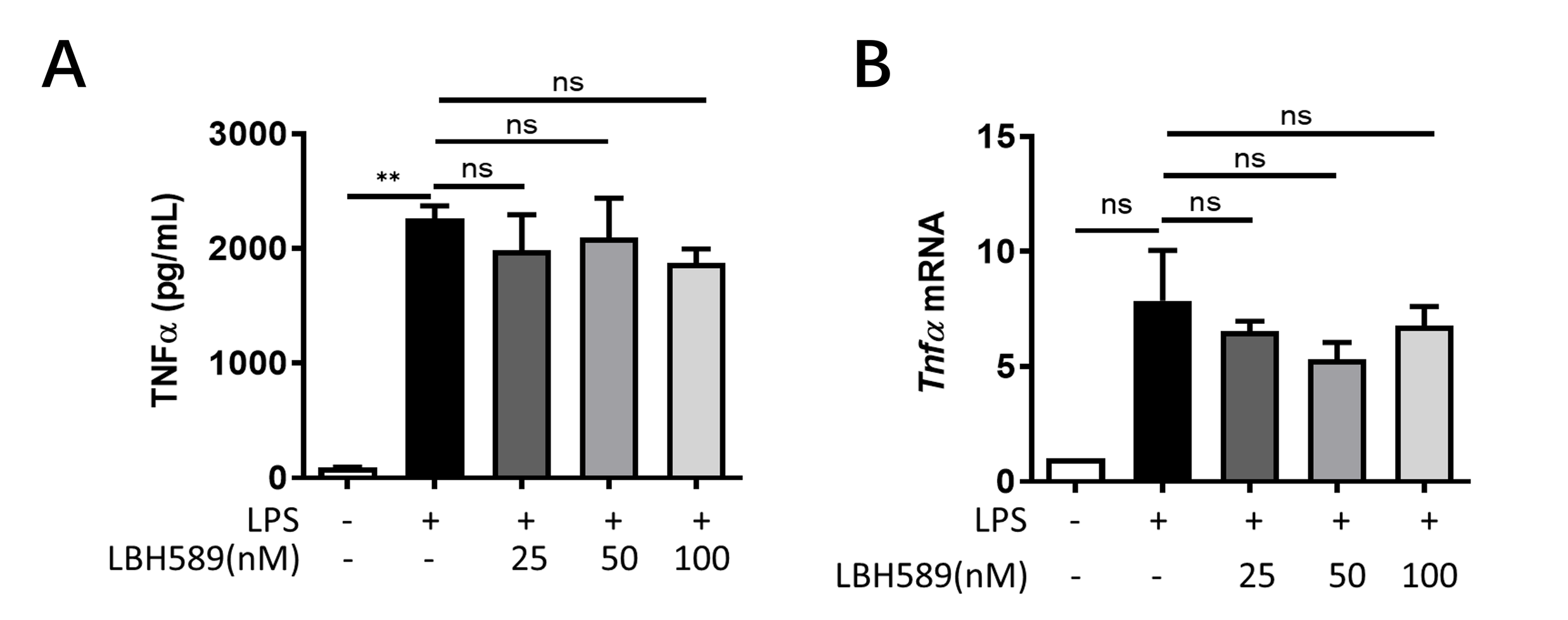

Supplement: S1 Fig — (A, B) J774A.1 cells were primed with LPS (1 µg/ml) for 6 h with or without LBH589. Supernatants were analyzed by ELISA for TNFα release in (A). The mRNA levels of Tnfα were detected by RT-qPCR in (B). Results are shown as mean ± SEM (n = 3). **indicates p < 0.001, ns not significant. (TIF) [file pone.0328522.s001.tif]

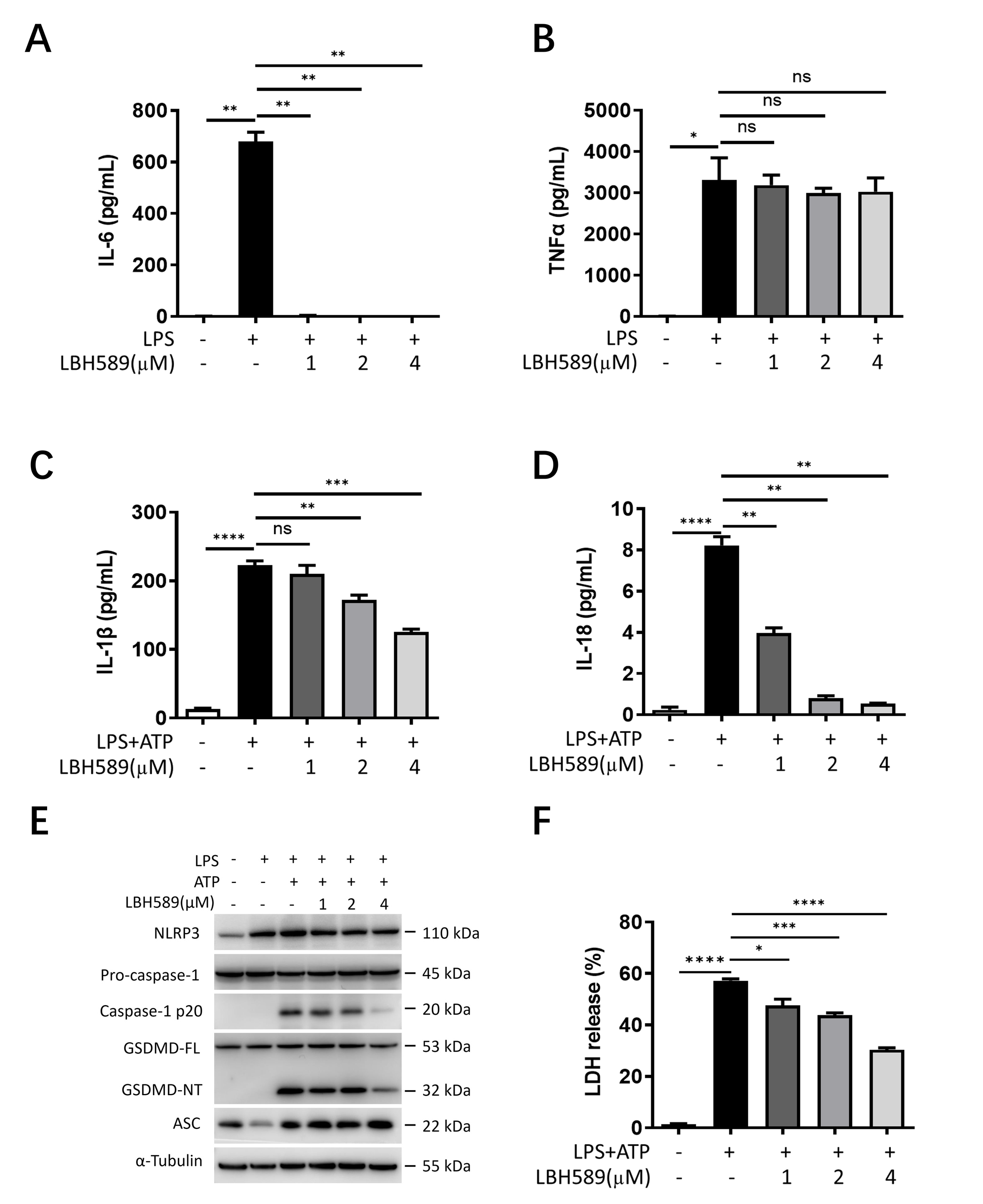

Supplement: S2 Fig — (A-D) BMDMs were treated with LPS (100 ng/ml) in the presence or absence of LBH589 (1, 2, 4 µM) for 4 h and minus or plus ATP (4 mM) for 1 h. The release of IL-6, TNFα, IL-1β and IL-18 in the supernatants were measured by ELISA. (E, F) LPS-primed BMDMs were stimulated with ATP with or without LBH589. Cell extracts were analyzed by immunoblotting to NLRP3, caspase-1, GSDMD, ASC, α-Tubulin served as a loading control. Supernatants for LDH release assay are shown in (F). Results are shown as mean ± SEM (n = 3). *indicates p < 0.05, **indicates p < 0.01 and ***indicates p < 0.001, ****indicates p < 0.0001. (TIF) [file pone.0328522.s002.tif]

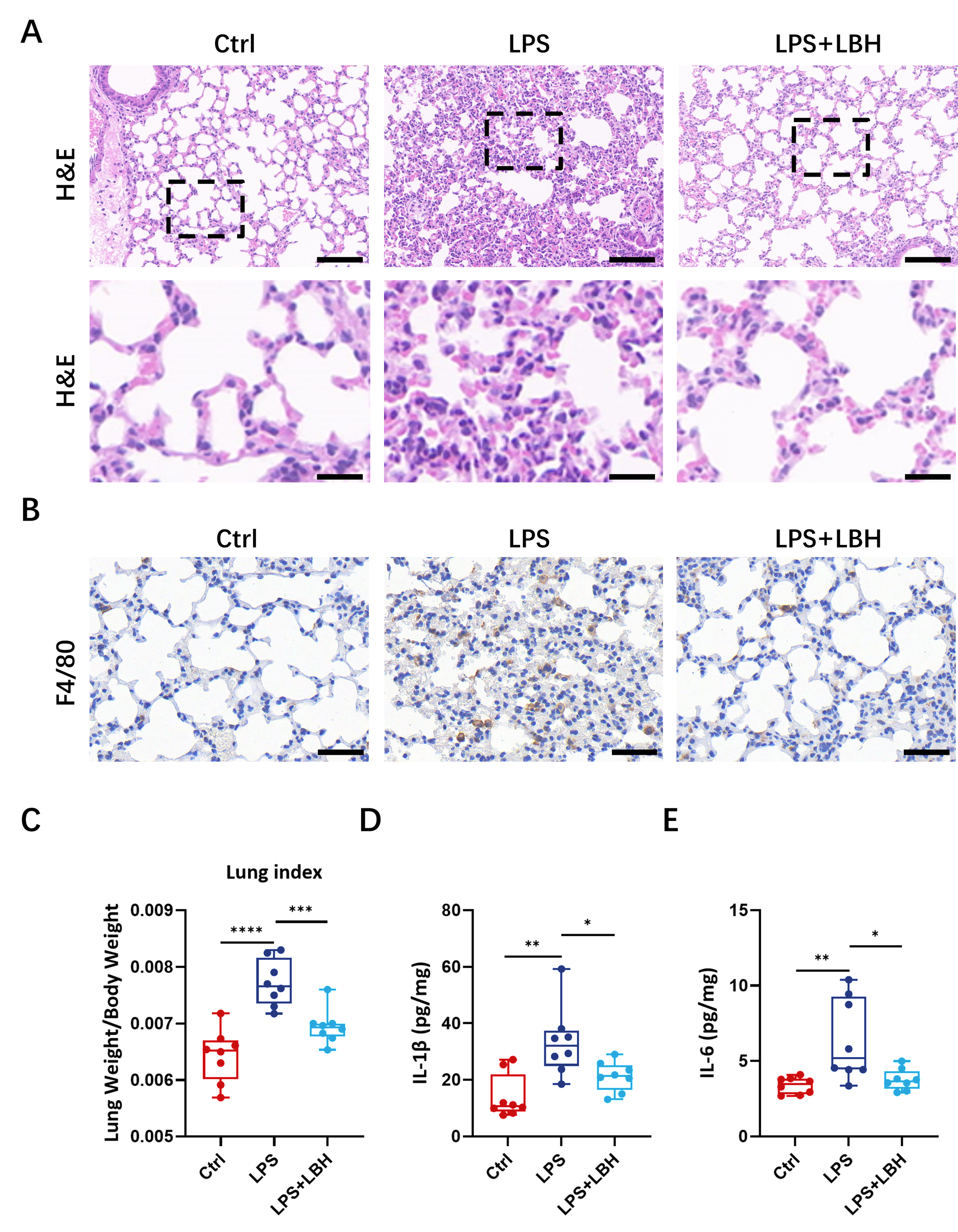

Supplement: S3 Fig — (A) H&E of lung tissue from mice that were pretreated with vehicle or 10 mg/kg LBH589 for three consecutive days and challenged with 5 mg/kg LPS 24 h prior tissue collection. Scale bar, 100 μm and 25 μm. n = 8 mice per group. (B) Lung sections from above mice were stained with F4/80 antibodies. Scale bar, 50 μm. (C) The lung index was calculated as lung weight/body weight and graphed as mean ± SEM. IL-1β (D) and IL-6 (E) concentrations in lung tissue from above mice were measured by ELISA. * indicates p < 0.05, **indicates p < 0.01, ***indicates p < 0.001, ****p < 0.0001. (TIF) [file pone.0328522.s003.tif]
