# Supplementary material for: LBH589 reduces oxidized mitochondrial DNA and suppresses NLRP3 inflammasome activation to relieve pulmonary inflammation
Source: PLoS One. 2025 Aug 4;20(8):e0328522. doi: 10.1371/journal.pone.0328522 (PMC12321101; doi:10.1371/journal.pone.0328522)
Supplement: S1 Text — (DOCX) [file pone.0328522.s004.docx]

**BMDM culture and stimulation**

Male C57BL/6J mice (4-5 weeks old) were purchased from Vital River Laboratories (Beijing, China), all protocols were approved by the ethics committee of the Beijing Institute of Transfusion Medicine (application number: IACUC-DWZX-2024-565). To acquire bone marrow-derived macrophages (BMDMs), the femur and tibia were collected from C57BL/6J mice, and bone marrow cells were flushed with PBS containing antibiotics and the cell suspensions were filtered through a 40-μm cell strainer for the removal of any cell clumps. Single-cell suspension was then cultured in complete DMEM with 100 units/mL penicillin-streptomycin (Solarbio, Beijing, China) and 50 ng/mL M-CSF (Peprotech, Rockford, USA) at 37°C. For full differentiation of BMDMs, the Cells were cultured for an additional 7 days with replacement of the medium every 3 days. The BMDM purity was assessed by flow cytometry using F4/80 (biolegend, California, USA) antibodies and was routine>95%.

BMDMs were cultured at 37℃ with 5% CO_2_. NLRP3 inﬂammasome activation was induced after 4 h priming with ultrapure LPS (100 ng/ml) by challenge with the NLRP3 activators ATP (4 mM) for 1 h. Afterward, the supernatants were collected for ELISA and LDH assay, while the whole cells were harvested for western blot analysis.
