# Supplementary material for: LBH589 reduces oxidized mitochondrial DNA and suppresses NLRP3 inflammasome activation to relieve pulmonary inflammation
Source: PLoS One. 2025 Aug 4;20(8):e0328522. doi: 10.1371/journal.pone.0328522 (PMC12321101; doi:10.1371/journal.pone.0328522)
Supplement: S1 Raw Images — (PDF) [file pone.0328522.s005.pdf]

Figure_1G

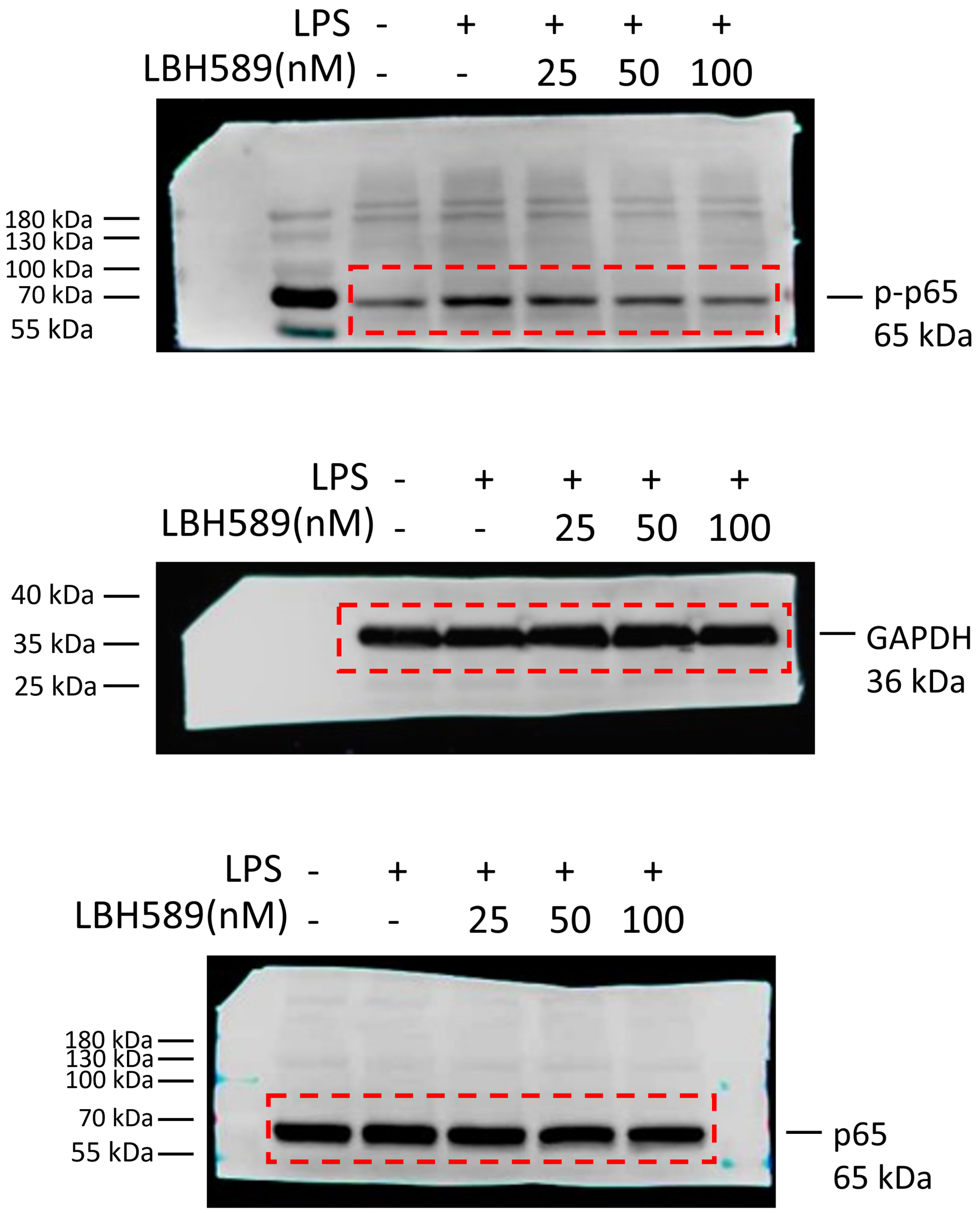

LPS - + + + +
LBH589(nM) - - 25 50 100
180 kDa
130 kDa
100 kDa
70 kDa
55 kDa
p-p65
65 kDa
LPS - + + + +
LBH589(nM) - - 25 50 100
40 kDa
35 kDa
25 kDa
GAPDH
36 kDa
LPS - + + + +
LBH589(nM) - - 25 50 100
180 kDa
130 kDa
100 kDa
70 kDa
55 kDa
p65
65 kDa

Figure_2A

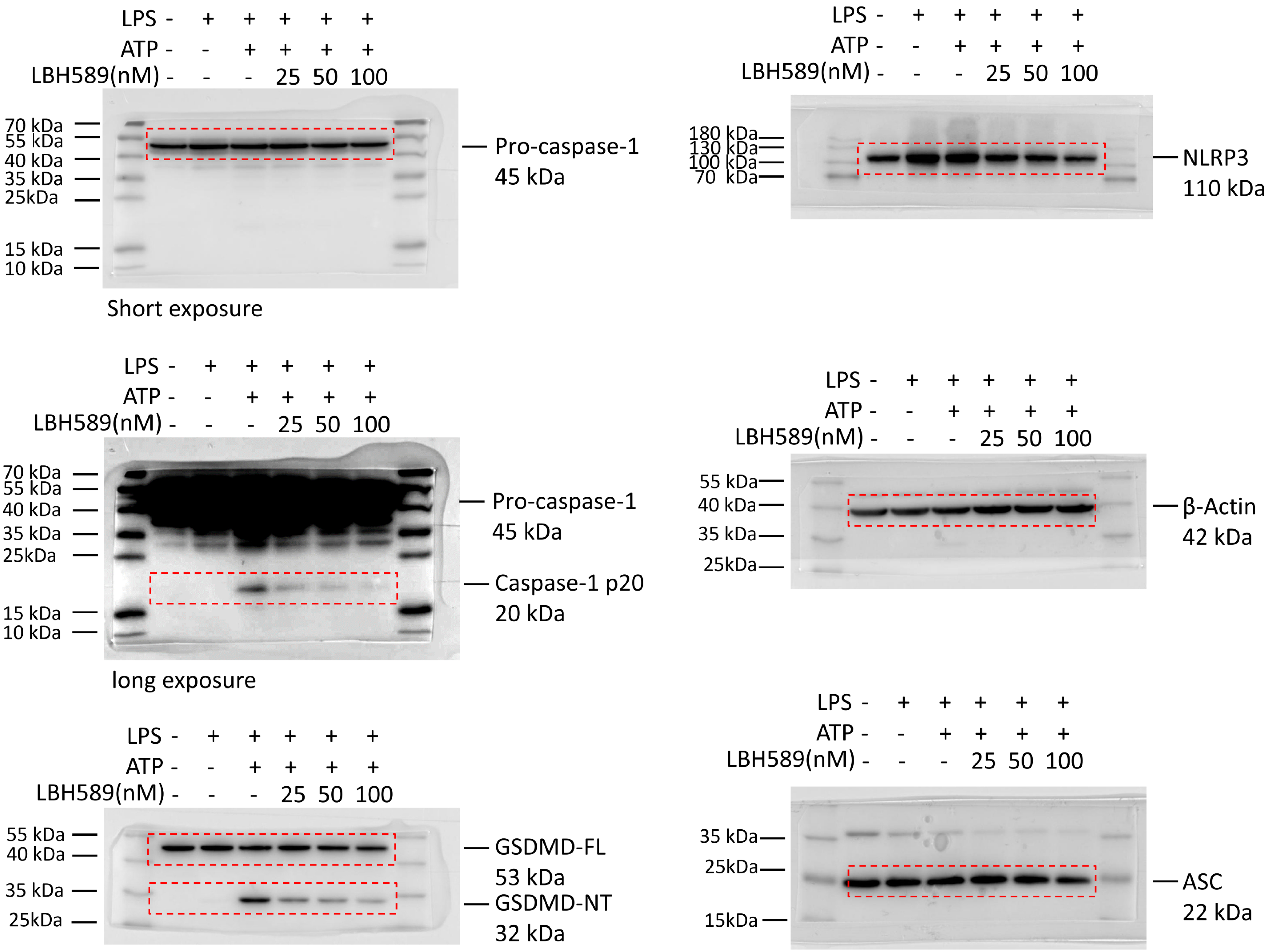

LPS - + + + + +
ATP - - + + + +
LBH589(nM) - - - 25 50 100
70 kDa
55 kDa
40 kDa
35 kDa
25kDa
15 kDa
10 kDa
Pro-caspase-1
45 kDa
Short exposure
LPS - + + + + +
ATP - - + + + +
LBH589(nM) - - - 25 50 100
180 kDa
130 kDa
100 kDa
70 kDa
NLRP3
110 kDa
LPS - + + + + +
ATP - - + + + +
LBH589(nM) - - - 25 50 100
70 kDa
55 kDa
40 kDa
35 kDa
25kDa
15 kDa
10 kDa
Pro-caspase-1
45 kDa
Caspase-1 p20
20 kDa
long exposure
LPS - + + + + +
ATP - - + + + +
LBH589(nM) - - - 25 50 100
55 kDa
40 kDa
35 kDa
25kDa
β-Actin
42 kDa
LPS - + + + + +
ATP - - + + + +
LBH589(nM) - - - 25 50 100
55 kDa
40 kDa
35 kDa
25kDa
GSDMD-FL
53 kDa
GSDMD-NT
32 kDa
LPS - + + + + +
ATP - - + + + +
LBH589(nM) - - - 25 50 100
35 kDa
25kDa
15kDa
ASC
22 kDa

Figure_4C

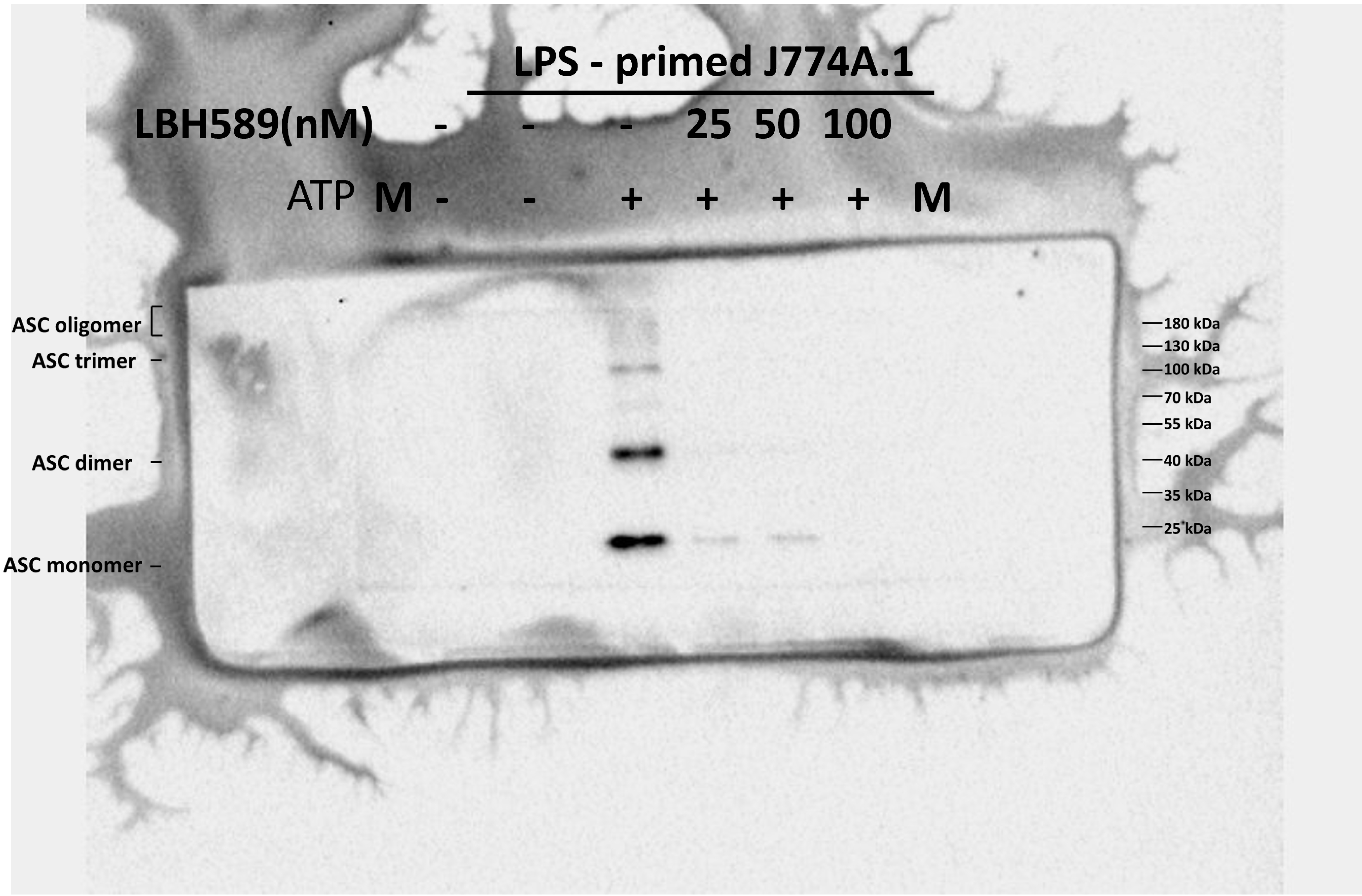

LPS - primed J774A.1
LBH589(nM) - - - 25 50 100
ATP M - - + + + + M
ASC oligomer
ASC trimer
ASC dimer
ASC monomer
180 kDa
130 kDa
100 kDa
70 kDa
55 kDa
40 kDa
35 kDa
25 kDa

Figure_5F

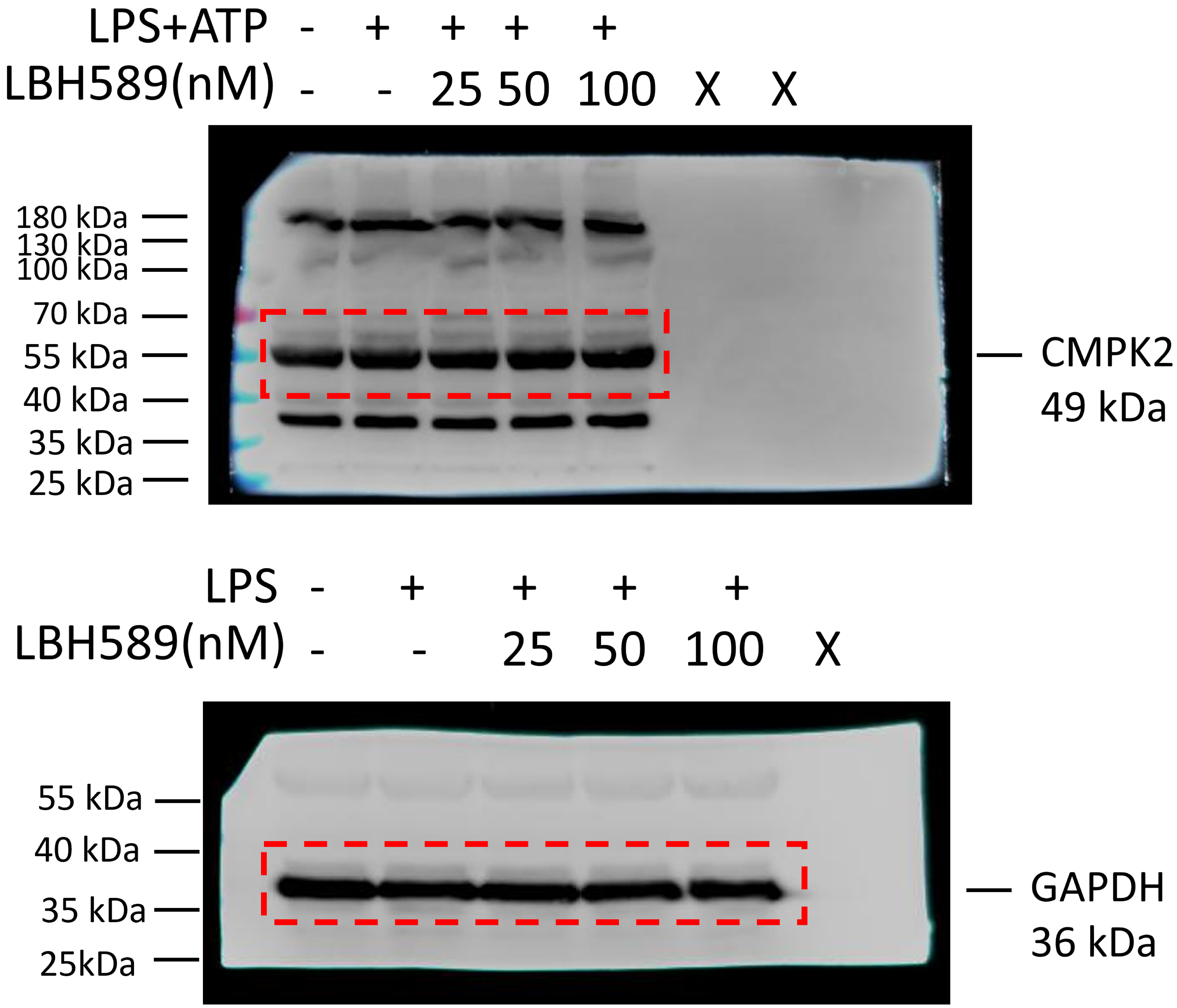
LPS+ATP - + + + +
LBH589(nM) - - 25 50 100 X X
180 kDa
130 kDa
100 kDa
70 kDa
55 kDa
40 kDa
35 kDa
25 kDa
CMPK2
49 kDa
LPS - + + + +
LBH589(nM) - - 25 50 100 X
55 kDa
40 kDa
35 kDa
25kDa
GAPDH
36 kDa

Figure_S1E

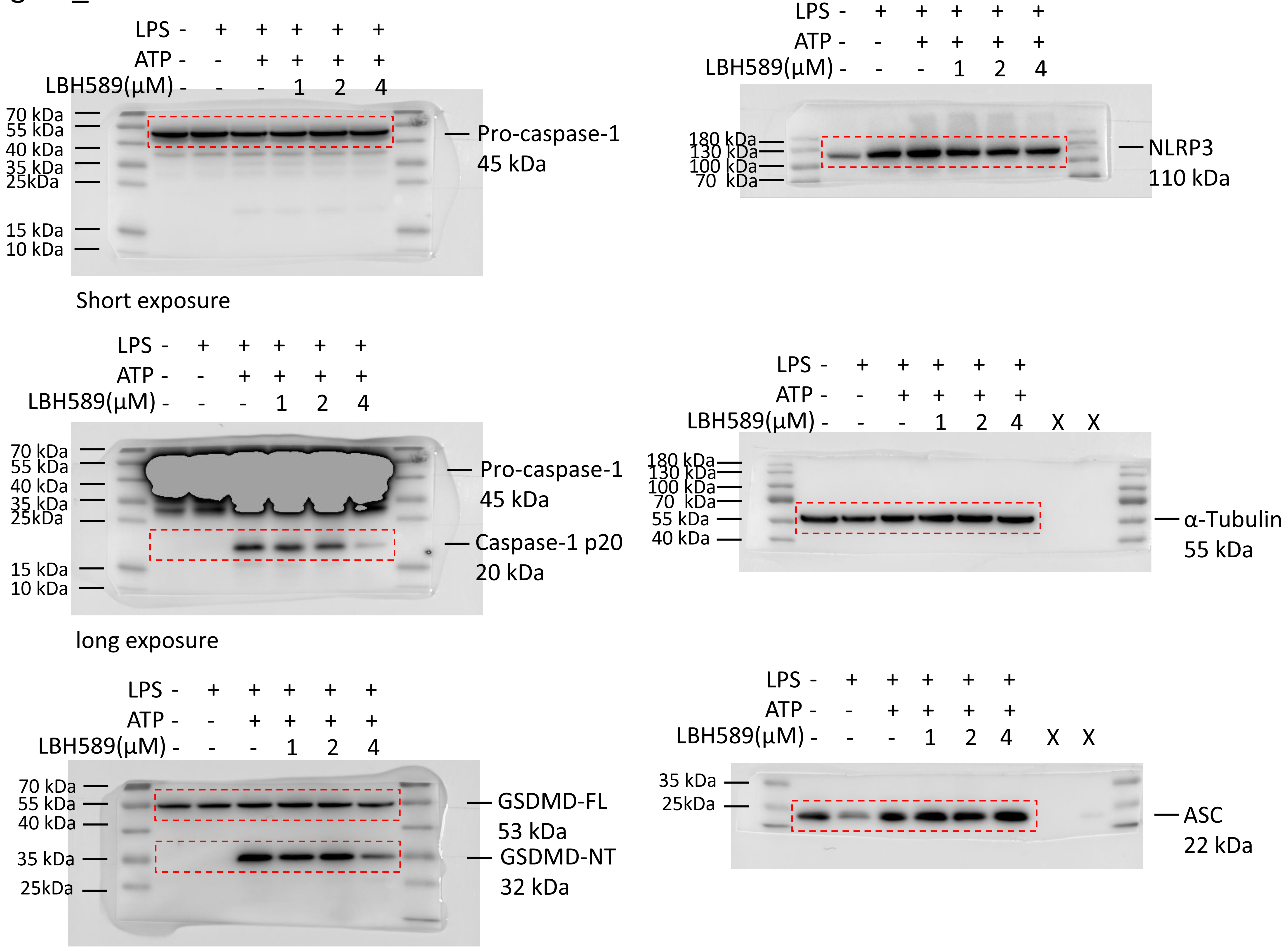

LPS - + + + + +
ATP - - + + + +
LBH589(μM) - - - 1 2 4
70 kDa
55 kDa
40 kDa
35 kDa
25kDa
15 kDa
10 kDa
Pro-caspase-1
45 kDa
Short exposure
LPS - + + + + +
ATP - - + + + +
LBH589(μM) - - - 1 2 4
180 kDa
130 kDa
100 kDa
70 kDa
NLRP3
110 kDa
LPS - + + + + +
ATP - - + + + +
LBH589(μM) - - - 1 2 4
70 kDa
55 kDa
40 kDa
35 kDa
25kDa
15 kDa
10 kDa
Pro-caspase-1
45 kDa
Caspase-1 p20
20 kDa
long exposure
LPS - + + + + +
ATP - - + + + +
LBH589(μM) - - - 1 2 4 X X
180 kDa
130 kDa
100 kDa
70 kDa
55 kDa
40 kDa
α-Tubulin
55 kDa
LPS - + + + + +
ATP - - + + + +
LBH589(μM) - - - 1 2 4
70 kDa
55 kDa
40 kDa
35 kDa
25kDa
GSDMD-FL
53 kDa
GSDMD-NT
32 kDa
LPS - + + + + +
ATP - - + + + +
LBH589(μM) - - - 1 2 4 X X
35 kDa
25kDa
ASC
22 kDa
